# SynchroLINNce: Toolbox for Neural Synchronization and Desynchronization Assessment in Epilepsy Animal Models

**DOI:** 10.21500/20112084.7329

**Published:** 2024-07-25

**Authors:** Sofa M. A. F. Rodrigues, Vinícius R. Cota

**Affiliations:** 1 Centro Universitário de Lavras, Lavras, Minas Gerais, Brasil. Centro Universitário de Lavras Centro Universitário de Lavras Lavras Minas Brazil; 2 Rehab Technologies - INAIL Lab, Istituto Italiano di Tecnologia, Genova, Italy. Rehab Technologies - INAIL Lab Istituto Italiano di Tecnologia Genova Italy; 3 Laboratório Interdisciplinar de Neuroengenharia e Neurociências (LINNce), Departamento de Engenharia Elétrica, Universidade Federal de São João del-Rei, São João del-Rei, Brasil. Universidade Federal de São João del-Rei Laboratório Interdisciplinar de Neuroengenharia e Neurociências (LINNce) Departamento de Engenharia Elétrica Universidade Federal de São João del-Rei São João del-Rei Brazil

**Keywords:** Toolbox, MATLAB, Epileptiform Spike, Epilepsy, Neural Synchronization, Caja de herramientas, MATLAB, espiga epileptiforme, epilepsia, sin cronización neuronal

## Abstract

Epilepsy is a worldwide public health issue, given its biological, social, and economic impacts. Considering several open questions about synchronization and desynchronization mechanisms underlying epileptic phenomena, the development of algorithms and computational toolboxes for such analysis is highly relevant to their research. Moreover, given the recent developments of neurotechnology for epilepsy, it is essential to understand that proposals like computational tools may provide consistent data for closed-loop control systems, necessary in neuromodulation treatment alternatives, and for real-time monitoring systems to predict the occurrence of epileptic seizures. In the present work, SynchroLINNce, a freely distributable MATLAB toolbox designed to be used by epilepsy neuroscientists, including software-untrained), is proposed. Among its features, several functionalities such as recording visualization, digital filtering, and correlation analysis, as well as more specific methodologies, such as mechanisms for the automatic detection of epileptiform spikes, morphology analysis of these spikes, and their coincidence between channels are presented.

## Resumen

La epilepsia es un problema de salud pública mundial debido a sus impactos biológicos, sociales y económicos. Considerando varias preguntas abiertas sobre los mecanismos de sincronización y desincronización que subyacen a los fenómenos epilépticos, el desarrollo de algoritmos y toolboxes computa- cionales para dicho análisis es altamente relevante para su investigación. Además, dado el desarrollo reciente de la neurotecnología para la epilepsia, es esencial entender que propuestas como las herramientas computacionales pueden proporcionar datos consistentes para sistemas de control en bucle cerrado, necesarios en alternativas de tratamiento de neuromodulación, y para sistemas de monitoreo en tiempo real para predecir la ocurrencia de crisis epilépticas. En el presente trabajo, se propone SynchroLINNce, una toolbox de MATLAB de distribución libre, diseñada para ser utilizada por neurocientíficos especializados en epilepsia (incluidos aquellos sin formación en software). Entre sus características, se presentan varias funcionalidades, como la visualización de grabaciones, el filtrado digital y el análisis de correlación, así como metodologías más específicas, como mecanismos para la detección automática de picos epileptiformes, el análisis de morfología de estos picos y su coincidencia entre canales.

## 1. Introduction

The first observation of neural bioelectricity, which oc curred through the detection of currents in the brains of rabbits and monkeys, dates back to 1875. However, the first human electroencephalogram (EEG) was recorded by Berger only 50 years later (Kiloh et al., 2013). Cur rently, there are many ways to record neural activity, and the choice of method is intrinsically connected to the type of analysis to be carried out and its objectives. For local field potential (LFP), a type of intracranial EEG to collect information from neuronal populations, extract ing features, offers significant possibilities for both phys iological (Ahmadi et al., 2021; Jackson & Hall, 2016) and pathological studies (Kiloh et al., 2013).

Over the past few years, several algorithms and tool boxes have been proposed in the literature to process neural bioelectricity recordings, facilitating feature ex traction for epilepsy studies. These tools include as sessments of processes like neural synchronization and desynchronization, which occur during pre-ictal, ictal, post-ictal, and interictal periods (Jiruska et al., 2013). The synchronous firing of neuronal populations asso ciated with pathological scenarios can generate easily distinguishable electrographic patterns, such as epilep tiform spikes. These electrographic patterns are rec ognized hallmarks of epilepsy (Niedermeyer, 2011) and represent common types of epileptiform paroxysms fre quently studied in this neurological disorder, produced by the neurophysiological involvement of excessively syn chronized potential firings (Bromfield et al., 2006).

In practice, pattern analyses involving epileptiform spikes have provided acquaintance about fundamental issues of excitability and neural circuitry in ictogene- sis and epileptogenesis processes (Moraes et al., 2021), setting up mechanisms for evaluating alternative treat ments for the disorder, which includes patients without a satisfactory response to traditional treatment methods (Tang et al., 2017; West et al., 2019). These new possi bilities include electrical stimulation protocols (Cota et al., 2009, 2023; Moraes et al., 2021), for example, and the development of closed-loop control systems, also fre quently aimed to establish early prediction of epileptic seizures (Carvalho et al., 2020; Kuhlmann et al., 2018; Stirling et al., 2021). By analyzing the state-of-art in toolboxes for the last twenty years, according to the cho sen research methodology that will be described later, it is possible to infer that the analyses, as those mentioned, are more often related to four basic types of develop ments. These are toolboxes for general analyses of EEGs and other types of recordings; standalone use method ologies, directed to epilepsy investigations or not, for internal use only or disseminated in the literature; spe cific toolboxes for epilepsy studies and multidisciplinary toolboxes for signal processing and artificial intelligence techniques, for example. Except for the last group, a sig nificant portion of these tools reported in the literature is developed for human recordings and, in some cases, used for epilepsy animal models.

In the first group, options like EEGLAB and more recent proposals such as Open Ephys and ripple-AI, can be highlighted. The first one was developed in MAT LAB, it is widely used for different neurological disor der studies and has new associated toolboxes, estab lished as plugins, and developed for epilepsy investiga tions (Delorme & Makeig, 2004). The second one, also a MATLAB proposal, was developed with open hard ware and freely distributable software for experimental electrophysiology, and like the previous one, it has been encouraging the growth of plugin development over the past three years (Siegle et al., 2017). The third op tion is a Python toolbox and demonstrates the trends for coming years of programming languages in this sce nario. It is focused on LFP analysis for neurophysiolog ical or non-neurophysiological detection of signals from the hippo campus, such as sharp wave ripples, using Ma chine Learning techniques (Navas-Olive et al., 2023).

Additionally, the specific toolbox group can be subdi vided into plugin toolboxes and independent proposals. In this first case, options such as EPINETLAB and eMI can be mentioned, using Spectral Kurtosis and Wavelet Transform techniques for high-frequency oscillations de tection and possible visualizations of epileptic seizure initiation zones (Jurkiewicz et al., 2021; Quitadamo et al., 2018). eMI uses a new Modulation Index (Tort et al., 2010) version aiming to mitigate spurious results of phase-amplitude coupling (Jurkiewicz et al., 2021).

In the group of specific toolboxes as standalone solu tions, there are MATLAB developments such as EPILAB and MIA. EPILAB was designed as a multifaceted feature extraction tool for seizure prediction studies, while MIA provides a centralized analysis type using best practices and general tools for neuroscientists within the frame work of epilepsy studies (Dubarry et al., 2022; Teixeira et al., 2011). For this tool, it is also relevant to mention the choice to provide multiple types of analysis for EEGs and electrocardiograms, such as average phase coherence and HR (Infinite Impulse Response) filters (Teixeira et al., 2011). In addition to the mentioned options, there is a group of epilepsy-specific toolboxes with resources for the imaging exam analyses providing significant inputs to the surgery-case treatments, for example. Among these are FocusDET (Marti Fuster et al., 2013) and moviEEG (Wong et al., 2016), both in MATLAB, and BrainQuake, a Python toolbox capable of providing parameters about the location of seizure initiation foci (Cai et al., 2022).

The last group, formed of multidisciplinary solutions, includes some of the most common examples in the lit erature for signal processing, such as the MATLAB Sig nal Processing Toolbox, which provides tools like the Fast Fourier Transform; data science libraries, such as Pandas in Python; and software for statistical analyses. There is also a growing use of nonlinear techniques: Lya punov exponents for the assessment of electrographic signatures like sharp waves (Yakovleva et al., 2020) and techniques derived from fuzzy entropy for the detection of typical EEG periods (Chiang et al., 2019).

In light of this scenario, this article introduces the toolbox SynchroLINNce, a freely distributable software with a modular structure designed to enhance the user experience. This solution was primarily aimed at neuro science laboratories with a multidisciplinary approach, serving professionals with limited or no programming knowledge. So, here we attend a spectrum of analyses, ranging from general solutions like data visualization, digital filtering, and correlation analysis to more spe cialized tools for neural synchronization analyses. The toolbox includes three methodologies proposed and pre viously validated by the authors: an automatic detec tion method for epileptiform spikes, an approach for spike morphology analysis, and a coincidence analysis method comparing different neural substrates.

## 2. Methods

Before introducing this section, it is relevant to remind that the SynchroLINNce toolbox was developed in MAT LAB as a freely distributable solution, and it is available under this link: https://github.com/sofiamafrodrigues/ SynchroLINNce. This section is divided into three parts: the development of signal processing methodologies, the presentation of the modular structure, interfaces, and other algorithms, and the chosen method to obtain the state of the art in similar toolboxes.

### 2.1 Signal Processing Methods

In order to develop quantitative and qualitative anal yses for the assessment of neural synchronization and desynchronization processes important in epileptic phe nomena, the SynchroLINNce toolbox was conceived to provide six main functionalities, defined after a thorough assessment of its signal processing context (Bromfield et al., 2006; Jiruska et al., 2013). Its set of methodologies (proposed or chosen from literature) was defined as tool box functionalities to enable different data outputs. Each functionality can be used individually or in combinations, depending on the intended analysis topics, along with a mechanism for automatic recording visualization.

Moreover, the proposed methods for automatic spike detection, spike morphology, and spike coincidence have been validated by evaluating classifier methodologies and using different epilepsy animal models. These analyses include assessments of the neural synchronization and desynchronization processes for these models and the feature extraction in an automatic recording classifica tion schema, for a potential seizure prediction model (De Oliveira et al., 2019; Rodrigues et al., 2019; Santos et al., 2021).

#### 2.1.1 Automatic Spike Detection

The first developed functionality was a method for au tomatic epileptiform spike detection, based on the defi nition that these electrographic signatures are transient events with amplitude above the recording baseline, sharp in shape, with a duration between 20 and 80 ms, asym metric amplitude increase, and followed by a slow wave and an amplitude decline (Gloor, 1975; Niedermeyer, 2011). In practice, this detection process was established using three types of thresholds involving user inputs as the desirable recording time for analysis. The first one is a moving amplitude threshold where the user defines a value between 0 and 100%, and a voltage amplitude value is calculated with this percentage based on the maximum amplitude observed in the recording during each one sec ond analysis window. Thus, if the user chooses 60%, and the maximum amplitude within the window is 1 mV, the threshold during that 1-second window will be set at .6 mV, and oscillations above this amplitude in the window are detected. For each 1-sec time window, the threshold voltage is thus recalculated to cope with the consider able variation of epileptiform spikes, in contrast to what happens with neuronal spikes (Rodrigues et al., 2019).

The second threshold sets a fixed amplitude value to ensure minimum acceptable spike amplitudes. Finally, the third is a temporal threshold, establishing the mini mum allowed distance between two consecutive epilepti form spikes. These two last parameters also rely on the user input and have a configuration set to avoid spuri ous results like counting polyphasic spikes as separate entities and to enhance the distinction of electrographic signatures from recorded baseline activity. At the end of the detection process, the following parameters are pro vided in addition to the number of detected spikes: max imum, average, and minimum amplitude values of these spikes; maximum, average, and minimum distances be tween them and their firing rate.

#### 2.1.2 Digital Filtering

Additionally, after conducting preliminary validation tests on this methodology to assess the efficiency of automatic detection compared to the same process performed by an expert electrophysiologist, it was observed that bet ter performance occurred in most cases with digitally filtered recordings in the frequency range of 10 to 100 Hz. Therefore, a digital filtering functionality was sub sequently developed, aiming to remove slow oscillations and high-frequency contents, undesirable for the pro posed spike detection.

Therefore, the automatic generation of filtered record ings was established in the chosen frequency range choos ing also between two types of digital filter methodologies: FIR (Finite Impulse Response) and HR. The FIR filter algorithm was developed using the windowing method, least squares approximation, and smoothing of the re sponse with applied windows using the Hanning func tion. For the IIR filter, a Butterworth-type digital fil ter was elaborated. However, in both cases, caution was necessary during filter application to avoid possible distortions leading to phase delays. Thus, the defined strategy was to filter in the forward direction, reverse the filtered sequence, and then apply this new sequence to the filter again.

#### 2.1.3 Spike Morphology

After developing the already presented functionalities to extract new information from spikes that would en able the micro and macrodomain analyses of neural sub strates, two additional types of methodologies were de veloped, structured as functionalities in the toolbox: epi leptiform spike morphology and ictal coincidence be tween neural substrates. The morphology analysis uses the slope measures calculated, which relate to the repre sentative average epileptiform spike extracted from the past detection data. These slope measures are divided into three average values: the rising segment of the spike, the spike descent segment, and the overall spike.

#### 2.1.4 Spike Coincidence

The Figure below presents a summary of this analysis. For coincidence analysis, a mechanism was established to evaluate neural interactions between substrates, com paring recordings from two different brain regions. One channel is designed as a reference and the other as the comparison channel, within a desired time window and using the previously saved time markers of the detected spikes. If a spike is found in reference and comparison channels within the time window, there is a coincidence. The percentage of coincident spikes in terms of the total analyzed period is provided:

**Figure 1:**
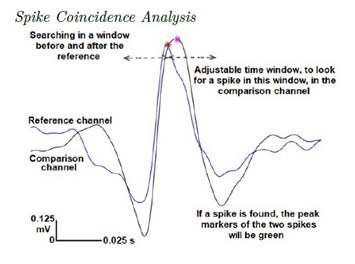
Spike Coincidence Analysis

#### 2.1.5 Correlation Analysis

With the consolidation of the mentioned functionalities, attending to the general purposes, it was embedded to SynchroLINNce a visualization mechanism and a corre lation functionality. In this latter case, it is a statistical analysis to compare two distinct neural substrates, gen erating a correlation coefficients matrix and a graphical output of this analysis. The chosen parameter was the Pearson coefficient (Fisher, 1970), defined for two arbi trary variables *A* and *B,* where *^a* and *^b* are the means of variables *A* and *B*, and *a A* and *ob* are their standard deviations. The scale will range from *-*1 to 1, with val ues indicating inverse and direct correlations at these extremes, and 0 when there is no linear interdependence:




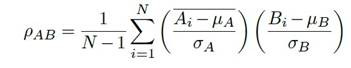




### 2.2 Toolbox Development

Although with some variation according to the signal processing method considered, a common standard de veloping for all and each of them was established. Ba sically, there is at least one GUI file (Graphical User Interface) in fig format, responsible for what the user sees and interacts with the software, and a .m format file algorithm, defining how the corresponding MATLAB function operates.

The first part of the toolbox thus consisted of devel oping the main structure for navigation between func tionalities: a .fig file with the interface structure config ured and the algorithm, a MATLAB function, for this central structure. In this algorithm, the functionalities are linked with its specific GUI structures, which will be presented later. With functions from the MATLAB GUI, each button is positioned and connected to the corresponding algorithm that responds to the user’s re quest. However, despite the independent modular struc tures defined for all components, this main structure was entirely completed when the others were finished, due to the mentioned connection mechanisms.

After defining the main structure, it was necessary to determine a strategy for receiving and saving experi mental data for each analysis. Thus, a mechanism was established in all toolbox functionalities, proposed to use for each new individual, except for morphology and spike coincidence analyses, due to the reuse of inserted spike detection data. For the individual initialization, the user will input information such as the experiment’s name, researcher, animal and group identification, num ber of recorded channels, sampling frequency, and am plification gain. These details were useful for all imple mented automation in the toolbox, for later extraction and verification purposes and to determine mechanisms for automatic saving.

As mentioned previously, some other specific items were included depending on the analysis tool. For in stance, in some cases it is relevant to inform whether the recordings were filtered or not, considering the influence of such a preprocessing step on the subsequent analysis. On the other hand, the mechanisms include replicable information, such as frequently repeated data like the sampling frequency and amplification gain. These struc tures allowed the user to proceed with the analysis with out entering non-essential information into the toolbox’s operation, streamlining data input. Every significant re sult for later visualization, according to the expected use of the toolbox, is automatically saved, either as a figure or a data structure from the current analysis. In these automatic saving mechanisms, regardless of the stored file type, an automatic structure was made considering the type of analysis, the name of the associated indi vidual, and, if applicable, the corresponding recorded channel, ensuring that information does not overwrite itself. Additionally, at possible moments of nomencla ture doubt or decision needed for automatic storage, the user must provide this decision with a command. For the figures, some other mechanisms for automatic sav ing in editable MATLAB format (.fig) and PNG were developed. For the data structures, it was established considering these are files in the typic MATLAB process ing format (.mat), divided between information directly related to experiment data and analysis.

As an example, to understand the structure of each functionality, here the automatic detection is presented. In this structure, each screen for user interaction via button commands and input information was defined by a function (a .m algorithm) and a GUI (a .fig file). Given the analysis characteristics, the need to improve interactivity, and of providing visualizing options to the operation, five screens were defined, with five distinct functions to control them. Within these functions, oth ers are called for data organization and to perform au tomatic spike detection. The same struct pattern was developed in the other functionalities to ensure modular ity and favor possible interventions, including additional functions and modules and potential future strategies such as embedded SynchroLINNce to other toolboxes. 2.3 Definition of the set of tools The tools to be included in the toolbox were defined after a bibliographic review aimed at investigating current and future trends for the coming years in the field of signal processing, especially for epilepsy assessments. An initial selection helped de fine the best strategies to develop the proposed tool box. Subsequently, a literature search was conducted to present the state of the art in this paper. Therefore, it was decided to analyze studies published in English and to create an initial list of articles obtained by entering the following keyword combinations into Google Scholar: “toolbox and epilepsy”, “software package and epilepsy”, “toolkit and epilepsy”, and “toolbox and LFP”. In the sequence, the articles from this list were filtered consider ing their relevance to the themes and excluding options without citations on the same research platform. Us ing this new list, with around 60 articles, the next step was to read and select the main options for comparison with the proposed toolbox, chosen based on the follow ing decreasing order of priority criteria: 1) the number of citations on Google Scholar; 2) quality of the detailing about the used strategies; and 3) overall writing quality. Additionally, searching for details about the use of tool boxes for animal models of epilepsy was also decided to complement the analysis. This process was carried out with one of the most widely used toolboxes in the field, EPILAB, with its Scholar Google citations, specifically regarding the use in recordings of epilepsy animal mod els. In practice, the searches were conducted looking for the term “animal” in each article that cited EPILAB, listed in Google Scholar.

## 3. Results

Results presented here are illustrated with real data ac quired in research carried out with animal experiments in our lab. The main graphical interface of the toolbox is shown below:

**Figure 2:**
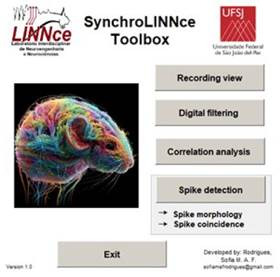
Main Interface of SynchroLINNce

**Figure 3:**
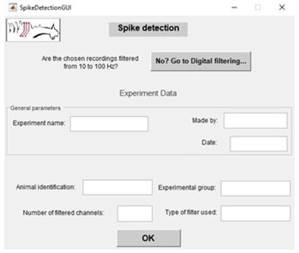
Interface for the User to Enter Experimental Data

The input of experiment information is done through interfaces like this example, useful for creating data struc tures as presented in the method section (see **Figure 3**).

### 3.1 Automatic Spike Detection

After entering experimental information, which can be accessed later or used as a data structure in MATLAB, the user inputs technical data such as the registered channel name, sampling frequency, and amplification gain. Providing the information, the user can access the main detection interface, which includes an automatic search for previously chosen recordings, their visualiza tion, and other possible required data. Additionally, there is also a display of specific information to guide the functionality’s use: a schematic demonstrating how the detection tool works, a text document containing the main global variables of the detection function, and function’s inputs and outputs.

**Figure 4:**
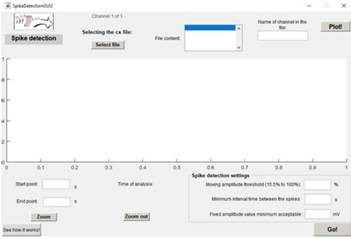
Interface for the User to Enter Information to Au tomatic Spike Detection

**Figure 5:**
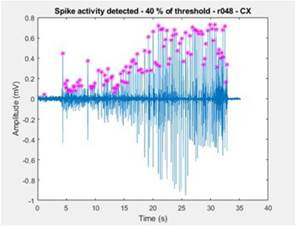
Results of the Automatic Spike Detection of the Example

To illustrate practical usage, an LFP recording from the cortex of the presented animal was chosen. The thresholds were input at 40% for the mobile threshold, a minimum time interval of .1 seconds, and a fixed voltage threshold of .1 mV (**Figure 4**). The detection result is presented in Figure 5. To proceed to another animal or new channels from the same animal, the user may close these screens. Besides the automatically saved detection data, there will be an automatic redirection to the pre ferred next step, as shown in de de Oliveira et al. (2019), Rodrigues et al. (2019), and Santos et al. (2021).

### 3.2 Spike morphology

**Figure 6:**
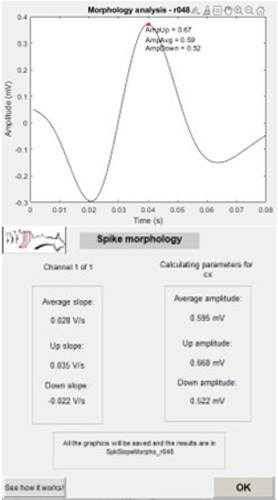
Results of the Spike Morphology Proposed Analysis of the Example

With the automatic detection results, it becomes possi ble to perform new analyses such as the functionalities of epileptiform spike morphology. Therefore, a new screen will appear, ending the detection analysis. If the user clicks on the spike morphology button, this analysis is automatically generated, which includes the two result screens presented in the panel (**Figure 6**).

The average spike considering the detected amount and the results of the set of proposed metrics are displayed. At this point, it is also possible to access a rep resentative figure about how the calculations are made and other technical information about the created MAT LAB function. By clicking “OK” at the end, the user is redirected to the same previous screen that led to this functionality and can directly proceed to the spike coin cidence functionality.

### 3.3 Spike Coincidence

The spike coincidence is independent of the morphology analysis, so the user will be directed to the following screen, requesting which channels to compare and the temporal window for coincidence. Here the user can ac cess the same technical information as in the previous functionalities, allowing to understand how this method works. The results of coincidence between the cortex and hippocampus for a .1 s window to the previous ani mal are shown in Figure 8.

**Figure 7:**
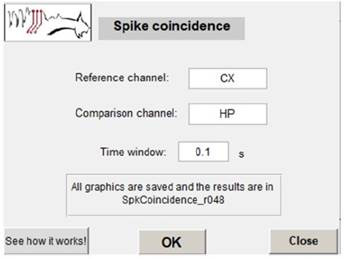
Interface for the user to Enter Information for Co incidence Analysis

If the user chooses to continue the analysis with other channels or windows, it will be necessary to fill out the information on the next screen. It is also allowed to return to morphology analysis or to the main interface using the exit buttons (**Figure 7**).

**Figure 8:**
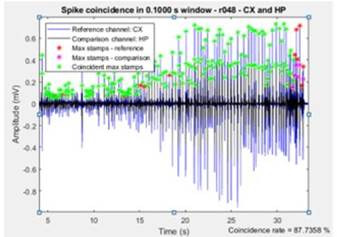
Coincidence Analysis between Cortex and Hippocampus of the Example

### 3.4 Digital Filtering

**Figure 9:**
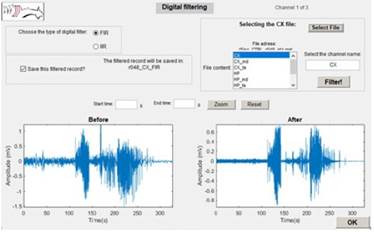
Interface for Entering Data to Digital Filtering and Visualization of the Filtered Recordings

For digital filtering, the user will select the raw recording jointly with the input information as in the detection mechanism, which type of filter, and to save or not the result. In this same interface, the user can view the raw and the filtered recordings and navigate with zoom on possible specific interest points. If there are other recorded channels from the same animal, by clicking OK, the user will be automatically directed to obtain these new filtered recordings (**Figure 9**).

### 3.5 Correlation Analysis

**Figure 10:**
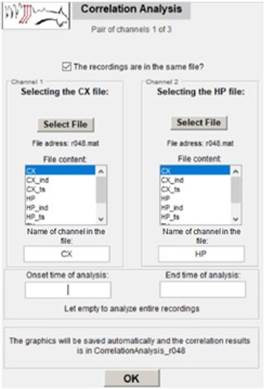
Interface for the User to Enter Information for Correlation Analysis

**Figure 11:**
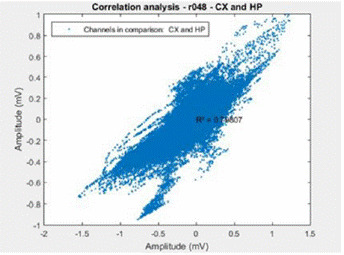
Correlation Analysis between Cortex and Hip pocampus of the Example

For correlation analysis, when the user clicks on the main menu in this functionality, the following self-explan atory interface is shown (see Figure 10). In this inter face, the user is also prompted to enter start and end times for the analysis, and such as the previous cases, the experiment information is automatically saved. By clicking OK, the correlation graphical result is gener ated. In this case, correlating cortex and hippocampus recordings of the same chosen animal are shown (see **Figure 10** and Figure 11).

## 4. Discussion

In this work, the SynchroLINNce toolbox was developed to provide mechanisms for evaluating the neural pro cesses of synchronization and desynchronization in LFPs from epilepsy animal models. It offers a combination of tools in the developed functionalities for epileptiform spike detection, spike morphology analysis, and spike coincidence evaluation. All the algorithms were tested on real data, as shown here, and results were also eval uated against other tools commonly used in the litera ture. Although not shown here for readability purposes, interested readers can refer to references De Oliveira et al. (2019), Rodrigues et al. (2019) and Santos et al. (2021). Furthermore, SynchroLINNce integrates gen eral functionalities for neuroscientists, such as record ing visualization, digital filtering, and correlation anal ysis. These general electrophysiological resources are well-suited for multidisciplinary professionals with little or no programming knowledge. For example, a user friendly interface was built to allow users to collect the desired data without understanding the intricacies of de velopment. These characteristics were achieved through the developed modular structure, which, in this set of tools, allows for the integration of new functionalities as well as with other existing platforms. Particularly, indexing data according to animal and channel identifi cation is useful for postprocessing of group values, such as in statistical analysis.

It is relevant to highlight that among previously avail able tools, only the eMI toolbox was originally developed for animal models. Moreover, by analyzing the Google Scholar citations of EPILAB, the specific toolbox option with the highest number of citations on this search plat form, it can be inferred that among the 104 citations, only one indicates its use for animal recordings. There is clear evidence that most developments are intended for human recording analysis. However, considering the prevalence and significance of epilepsy animal models for research, it is noteworthy that algorithms and toolboxes should be capable of handling potential peculiarities in these types of recordings despite the numerous similar ities already reported in the literature compared to hu man physiology (Kandratavicius et al., 2014).

Although not intended for usage with patient data, we believe that such application of SynchroLINNce tool box is feasible with minor effort. From the technical point of view, such as the necessity of new code or in terfaces, we believe not much would be necessary. For instance, the inclusion of fields for human patient data could be of great usefulness and a relevant topic to fu ture work. Developments like channel management tools could also be of importance, considering EEG recordings from patients usually have a significant channel count. Naturally, users processing human data need to first val idate findings by comparing them to benchmarks, once different signals, even when only slightly distinct, which can yield different and possibly invalid outcomes. Addi tionally, although the toolbox allows parameter adjust ments such as cutoff frequencies, which adds to its flexi bility and usability, we explicitly recommend not to use the tool for diagnosis purposes, as it may require proper validation and certification from sanitary organizations.

In any case, with the increasing use of open-source hardware and open-source software, it is well established that there is a significant gap in application-specific tool boxes and embeddable algorithms for larger signal pro cessing platforms (e.g., Open Ephys) in the context of epilepsy research. In fact, this growth is driven by gen eral technology development trends and more specific issues, such as the need for closed-loop systems to treat ment strategies (Stirling et al., 2021). There have been some specific strategies, especially in the last four years, but primarily focused on electrophysiology mechanisms in non-pathological contexts (De Sousa et al., 2022). Therefore, toolsets like the SynchroLINNce toolbox can be considered not only as standalone solutions, but also from a software integration perspective, with options like Open Ephys, given both are modular in structure.

It is well-known that a significant portion of patients who experience seizures are not effectively treated, a scenario that severely jeopardizes overall quality of life. On the other hand, there have been recent significant improvements in seizure detection and prediction algo rithms. Signal processing techniques, such as approaches involving EEG decomposition into different modes, can assist in diagnosis, seizure detection, prediction, and real- time event detection in long-term monitoring systems, using adaptive and data-dependent techniques (Carvalho et al., 2020). Additionally, the assessment of inter ictal spike rates can provide adequate mechanisms to gener ate specific algorithms for each patient (Kuhlmann et al., 2018). Furthermore, novel seizure prediction approaches are resulting from the exploration of machine learning techniques and susceptibility measures, such as physio logical biomarkers (Stirling et al., 2021). Analogously, with its simplified spike detection structure, as well as the morphology and coincidence analyses with low com putational cost, the proposed toolbox can also be used in real-time monitoring strategies with minimal adapta tions, similar to those presented.

Analyzing the literature, it is important to mention that there are alternative approaches for the automatic detection of epileptiform spikes, such as the choice to filter within a specific frequency range and to estab lish thresholds for spike peak detection (Janea et al., 2015). However, in this case, the thresholds were de termined from statistical distributions of the signal en velope. Complementarily, Reus et al. (2022) discuss the potential of automatic detection tools and the per sistent limitations in commercial proposals, emphasiz ing the ongoing development scenario. As previously discussed, these solutions can provide essential features for patient monitoring and seizure prediction strategies. For more details regarding the possibilities offered by this proposed detection and the functionality of mor phology and coincidence, we recommend the works De Oliveira et al. (2019), Rodrigues et al. (2019) and San tos et al. (2021).

Finally, although the toolbox was designed as a free distribution option, some users may have difficulties ob taining the MATLAB license, even though it is a com monly chosen software for research in the Neuroscience field. Therefore, for future works, the authors consider transferring these developed technologies to a fully open- source environment, such as Python.
